# Shorter Leukocyte Telomere Length Is Associated with Worse Survival of Patients with Bladder Cancer and Renal Cell Carcinoma

**DOI:** 10.3390/cancers13153774

**Published:** 2021-07-27

**Authors:** Xi Zheng, Felix Wezel, Anca Azoitei, Sabine Meessen, Wenya Wang, Gregoire Najjar, Xue Wang, Johann M. Kraus, Hans A. Kestler, Axel John, Friedemann Zengerling, Christian Bolenz, Cagatay Günes

**Affiliations:** 1 Department of Urology, Ulm University Hospital, 89081 Ulm, Germany; xi.zheng@uniklinik-ulm.de (X.Z.); felix.wezel@uniklinik-ulm.de (F.W.); anca.azoitei@uniklinik-ulm.de (A.A.); Sabine.Meessen@uniklinik-ulm.de (S.M.); Wenya.Wang@uniklinik-ulm.de (W.W.); gregoire.najjar@uniklinik-ulm.de (G.N.); Xue.Wang@uni-ulm.de (X.W.); axel.john@uniklinik-ulm.de (A.J.); Friedemann.Zengerling@uniklinik-ulm.de (F.Z.); Christian.Bolenz@uniklinik-ulm.de (C.B.); 2 Institute of Medical Systems Biology, Ulm University, 89081 Ulm, Germany; johann.kraus@uni-ulm.de (J.M.K.); hans.kestler@uni-ulm.de (H.A.K.)

**Keywords:** bladder cancer, renal cell carcinoma, leukocyte, telomere, telomerase

## Abstract

**Simple Summary:**

Intrinsic telomere shortening promotes tumorigenesis in cells with impaired DNA damage repair mechanisms, as dysfunctional telomeres lead to chromosomal instability. More recent data show that the telomere length of peripheral blood leukocyte (PBL) cells can be a prognostic marker for survival of patients with solid tumors. However, reports on bladder cancer (BC) and renal cell carcinoma (RCC) are not consistent and partly contradictory. Our results show, first, that telomere length is shorter in patients with BC or RCC compared to patients without malignant disease. More importantly, the relative telomere length (RTL) of PBL cells is associated with survival of patients with BC and RCC. Thus, telomere length in PBL cells could be an auxiliary prognostic marker in BC and RCC.

**Abstract:**

Background: Telomeres are protein–DNA complexes at the tips of linear chromosomes. They protect the DNA from end-to-end fusion and exonucleolytic degradation. Shortening of telomeric DNA during aging can generate dysfunctional telomeres, promoting tumorigenesis. More recent data indicate that both short and long telomeres of peripheral blood leukocyte (PBL) cells can serve as prognostic biomarkers for cancer risk and may be associated with survival of patients with solid cancers. Telomere length in PBL cells could also be a potential prognostic biomarker for survival in bladder cancer (BC) or renal cell carcinoma (RCC). Methods: The relative telomere length (RTL) of PBL cells was assessed in patients with BC (*n* = 144) and RCC (*n* = 144) by using qPCR. A control population of patients without malignant disease (NC, *n* = 73) was included for comparison. The correlation and association of RTL with histopathological parameters and overall survival (OS) were evaluated. Results: Patients with BC and RCC had significantly shorter telomeres compared to patients without malignant disease. Within the cancer cohorts, multivariate analysis revealed that short RTL is an independent predictor of worse survival in BC (*p* = 0.039) and RCC (*p* = 0.041). Conclusion: Patients with BC and RCC had significantly shorter telomeres compared to the normal population. Shorter RTL in BC and RCC was an independent predictor of reduced survival.

## 1. Introduction

Bladder cancer (BC) ranks as the tenth most common cancer throughout the world, with an estimated 549,000 new cases diagnosed in 2018, accounting for about 3% of all cancer diagnoses that year [1]. Kidney cancer is among the 10 most common cancers in European communities [2], with approximately 403,262 new cases and 175,098 deaths worldwide in 2018 [1], among which renal cell carcinoma (RCC) accounts for approximately 90% of malignant kidney tumors [2].

Patients with non-muscle-invasive BC (NMIBC) can be potentially cured by transurethral resection, BCG immunotherapy and intravesical chemotherapy. However, recurrence rates are high (60–70%) and approximately 30% show a progress to muscle-invasive BC (MIBC). About 25% of newly diagnosed BCs are MIBC [3]. Radical cystectomy (RC) is the treatment of choice in MIBC, however, overall survival rates after RC remain rather low, ranging between 40% and 60% after 5 years [4,5,6].

In RCC, about 30% of patients will develop metastatic disease despite initial curative surgical therapy [7]. Prognostic factors in BC and RCC are mainly based on clinicopathologic variables while there are still no molecular markers established for routine clinical use [8]. The identification of additional prognostic biomarkers that can be used in combination with clinical variables to predict their risk of recurrence and survival would improve counseling and treatment of these patients.

Previous genetic analyses and recent high-throughput sequencing results identified a large number of genetic mutations. These data show that along with mutations in TERT promoter, FGFR3 and TP53 pathways, the most frequent genetic alteration in BC is loss of heterozygosity of chromosome 9, including deletions at 9p or 9q [9,10,11,12,13,14,15]. In fact, loss of chromosome 9 p is also found in RCC, and Brunelli et al. [16] and Di Nunno et al. [17] reported that RCC patients harboring chromosome 9 p loss have worse clinical outcomes. However, there are still no established and common consensus biomarkers for BC and RCC prediction and prognosis.

Telomeres are protein–DNA complexes consisting of tandem repeats of TTAGGG at the tip of linear chromosomes that protect them from being recognized and processed following DNA breakage and exonucleolytic degradation, preventing the loss of genetic information [18,19]. Telomeric DNA has an average length of 5–15 kilobases (kb) and shortens around 100–200 base pairs (bp) per cell division in human somatic cells [20,21,22]. The shortening of telomere length during aging is mainly due to the downregulation of telomerase, a telomere-specific enzyme that extends telomeres in the majority of somatic cells [23,24]. In adult tissues, telomerase activity is restricted to certain normal cell types, including germ cells, stem cells or progenitor cell compartments of some tissues [25,26]. However, around 90% of human somatic carcinomas and approximately 99% of urothelial carcinomas of the bladder are telomerase positive, indicating that they retain the telomeres during the replication of tumor cells [24,25]. Previous reports indicated that telomerase activity could be detected in a large proportion (60–80%) of RCC but not corresponding normal kidney samples [27,28,29,30]. Reactivation of telomerase in bladder cancer is mainly due to TERT promoter mutations, but the mechanism for telomerase reactivation in RCC remains largely unknown [31,32,33]. In the absence of telomerase, a gradual loss of telomeric DNA in dividing somatic cells limits their proliferative capacity, resulting in replicative senescence, apoptosis, or neoplastic transformation, depending on the genetic background [25,34,35,36,37,38,39,40,41]. In line with experimental data, germline mutations in telomerase components and telomere binding proteins lead to shortened telomeres and increased tumor formation, with a reduced latency period at a young age in humans, supporting the hypothesis that telomere shortening does in fact represent a causal factor for tumor development in humans [42,43,44,45,46]. Telomere length abnormality was also suggested as a potential risk factor for BC and RCC [47,48,49,50,51].

Numerous observational studies have assessed the associations of leukocyte telomere length (LTL) with the risk of cancers, including BC and RCC [52,53]. Determination telomere length in peripheral blood leukocytes (PBL) could provide a non/low-invasive technique diagnostic and/or prognostic tool for cancer risk screening or survival prognosis. Interestingly, in contrast to the above-described associations of telomere dysfunction with tumor initiation, population-based studies have revealed that longer leukocyte telomeres can also be associated with an increased risk of tumor development in several tissues [54,55,56]. In support of this, Haycock et al. conducted a Mendelian randomization (MR) study, which showed that increased telomere length (TL) due to germline genetic variation was significantly associated with a higher risk of developing cancer, including BC (OR 2.19 [1.32–3.66]) and RCC (OR 1.55 [1.08–2.23]) [57]. Interestingly, recent studies also revealed that the TL of PBL cells has prognostic potential to predict survival of patients with various types of solid cancers [58,59,60,61,62,63,64,65,66]. However, inconsistent results were reported for distinct cancer types, both in the context of cancer risk assessment and survival prognosis; while some studies reported that longer PBL TL correlated with worse survival, i.e., in breast cancer [58], clear cell RCC [59], prostate cancer [60], and hepatocellular carcinoma [61], other reports indicated that shorter PBL TL correlated with worse survival in BC [62], gastric cancer [63], colorectal cancer [64,65], ovarian cancer and cervical cancer [66]. Importantly, it was shown that long and heterogeneous TL in blood lymphocytes indicated increased BC risk [67], while another study observed shorter PBL TL in BC patients [68]. For kidney cancer, telomere shortening in PB lymphocytes was reported to be a genetic predisposing factor for RCC [69], while another study indicated a strong association between longer PBL TL and an increased risk for developing RCC [70]. Similarly, while Callahan et al., reported an association between shorter PBL TL and poorer RCC disease-specific survival [71], Svenson et al. revealed that ccRCC patients with the longest blood cell telomeres (fourth quartile) had a significantly worse prognosis [59].

In order to add an independent set of information whether PBL TL is associated with cancer risk and patient survival, in this study we analyzed the correlation and association of RTL of PBL cells with histopathological parameters and overall survival (OS) of patients with BC (*n* = 144) and RCC (*n* = 144), and in a normal control population without malignant disease (NC, *n* = 73), by measuring the RTL in PBL cells by quantitative PCR. Our studies show that shorter PBL TL is associated with worse survival of patients with BC or RCC. In addition, our results show that TL is shorter in PBL cells of patients with RCC or BC compared to individuals without malignant disease, indicating that telomere shortening in PBL cells correlates with increased cancer risk.

## 2. Materials and Methods

### 2.1. Blood Samples and Clinical Data

The clinical information and follow-up data were obtained from the Department of Urology at the Ulm University Hospital and the Comprehensive Cancer Center Ulm. The study was performed from 2012 to 2018, and the follow-up duration of the cancer patient cohorts was 79 months for BC and 83 months for RCC. OS was defined as the period from the date of diagnosis to death or last follow-up.

Blood samples were obtained before surgery (and/or medical systemic therapy) from patients with bladder cancer (*n* = 144) or renal cell carcinoma (*n* = 144; including ccRCC, *n* = 120; papillary RCC, *n* = 17; chromophobe RCC, *n* = 7), or without malignant disease (control cohort), with informed written consent and local research ethics committee approval (442/17, 239/18). The NC group consisted of 73 patients without any history of cancer. The staging of BC was applied according to the TNM classification system from the Union for International Cancer Control (UICC, 2009) and the staging of RCC was applied according to the TNM classification system from the American Joint Committee on Cancer (AJCC, 2010). The grading of BC was applied according to the World Health Organization (WHO)/2004 and WHO/1973 classification. The grading of RCC was applied according to the WHO/2004 classification.

### 2.2. DNA Extraction

EDTA blood samples were mixed with 35 mL lysis buffer (Table S1) and incubated on ice for 30 min, followed at 161× *g* for 15 min. Cell pellets were then resuspended with 5 mL lysis buffer, and mixed and at 161× *g* for 15 min. This step was repeated 3 times. The cell pellet was then mixed with 5 mL SE buffer (Table S1), 50 µL Proteinase K (10 µg/µL), and 250 µL 20% SDS solution, and incubated overnight at 55 °C while shaking. The next day, 1.7 mL 6 M NaCl was added, and the mixture was at 2470× *g* at 25 °C for 15 min. The supernatant was transferred into a new Falcon tube, and visible DNA pellets were washed with 15 mL 100% EtOH, followed with 15 mL 75% EtOH, then diluted into 400 µL TE buffer (Table S1). The DNA solution was placed on the shaker overnight at room temperature and then stored at 4 °C.

### 2.3. Determination of RTL by Quantitative Polymerase Chain Reaction (qPCR)

PBL RTL was measured via qPCR as described in previous studies [72,73,74]. The standard curves for telomere standard and human 36B4 were generated by performing serial dilutions with an absolute telomere length assay [72]. Plasmid DNA (pBR322, Sigma-Aldrich, St. Louis, MO, USA) was added to each standard to maintain a constant 30 ng of total DNA per reaction tube (Figure S1). The oligomers of the standards were as follows: telomere standard: (TTAGGG)_14_; 36B4 standard: CAGCAAGTGGGAAGGTGTAATCCGTCTCCACAGACAAGGCCAGGACTCGTTTGTACCCGTTGATGATAGAATGGG. The primers (5′ to 3′) used for qPCR were CGGTTTGTTTGGGTTTGGGTTTGGGTTTGGGTTTGGGTT (Tel-F), GGCTTGCCTTACCCTTACCCTTACCCTTACCCTTACCCT (Tel-R), CAGCAAGTGGGAAGGTGTAATCC (36B4-F), and CCCATTCTATCATCAACGGGTACAA (36B4-R). For the PCR reactions, 384-well plates were used, and BJ, BJ-hTERT, and U2OS cells were included on each plate as calibrator DNAs for experimental control. Measurements of each sample (25 ng) were done in triplicate and the average value was calculated. The ratio of telomere copy number (T) to single gene copy number (S) was determined as T/S = 2^–^^ΔCT^, of which ΔCT = average CT _telomere_–average CT _single-copy gene(36B4)_. The RTL of each sample was normalized (defined) as the T/S values from the samples divided by the T/S values from the reference samples (BJ samples were used as reference DNA in this case). PCR amplification was performed with a ViiA 7 qPCR cycler (Applied Biosystems, Foster City, CA, USA) with the following running programs: one cycle at 95 °C for 15 min, followed by 40 cycles at 95 °C for 15 s and 54 °C for 1 min for telomere; one cycle at 95 °C for 15 min, followed by 40 cycles at 95 °C for 15 s, 58 °C for 20 s, and 72 °C for 30 s for 36B4. The median RTL of the BC and RCC groups was regarded as the cut-off for long and short RTL subgroups.

### 2.4. Statistical Analysis

SPSS software (SPSS, Inc., Chicago, IL, USA) was used for statistical analysis. Spearman’s correlation analysis was performed to investigate the correlation between age and RTL. The Mann–Whitney test was performed to evaluate RTL in different populations. One-way analysis of variance (ANOVA) was used to evaluate RTL among BJ, BJ-hTERT, and U2OS cells (multiple groupwise comparisons). Spearman′s correlation analysis, Pearson’s chi-squared test, and Pearson’s chi-squared test with Yates′s correction for continuity were performed to compare the differences between RTL and clinical characteristics in patient subgroups. Kaplan–Meier curves were generated for survival analysis with the log-rank test to compare the prognosis impact in RTL subgroups. Multivariate analysis was performed with Cox regression model to evaluate the hazard ratio and 95% confidence interval. The statistical significance was set as *p* < 0.05. For the *p*-values, a maximum of three significant digits are shown.

## 3. Results

### 3.1. Basic Charactersristics of the Study Population and Group Comparison

**Figure 1:**
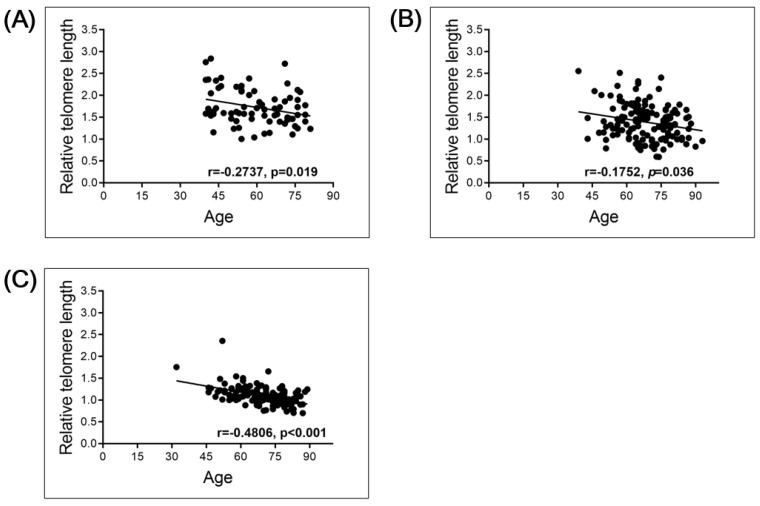
Linear correlation of RTL with age in normal control (NC), bladder cancer (BC), and renal cell carcinoma (RCC) groups: (**A**) NC group (*n* = 73); (**B**) BC group (*n* = 144); (**C**) RCC group (*n* = 144).

**Figure 2:**
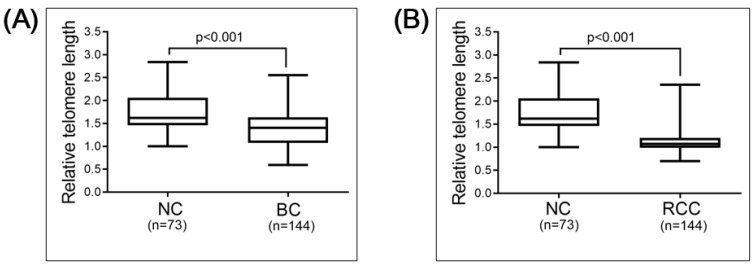
Comparison of RTL: (**A**) normal control (NC) group (*n* = 73) versus bladder cancer (BC) group (*n* = 144); (**B**) NC group (*n* = 73) versus renal cell carcinoma (RCC) group (*n* = 144). Comparisons were performed using Mann–Whitney test.

The total study population consisted of 73 individuals without malignant disease (normal controls, NC), 144 patients with bladder cancer (BC) and 144 patients with renal cell carcinoma (RCC; Table 1). Spearman’s correlation analysis revealed that the relative telomere length (RTL) negatively correlated with age, as expected, in the NC group (r = 0.2737, *p* = 0.019, *n* = 73), the BC group (r = 0.1752, *p* = 0.036, *n* = 144) and the RCC group (r = 0.4806, *p* < 0.001, *n* = 144; Figure 1). To investigate whether the RTL in the PBL of the NC group and the cancer patient groups differed, we compared the RTL in the NC (average age, 60 years) and BC (average age, 68 years) groups, and the NC and RCC (average age, 69 years) groups. The results show significantly longer RTL in the PBL of the NC than the BC (*p* < 0.001) or RCC (*p* < 0.001) group (Figure 2). This significance could be observed/stay in line within less sample numbers but more closely to the age-matched NC population, when compared with BC and RCC cohorts, though the sample numbers were lower (Figure S2).

### 3.2. Correlation between RTL and Clinical Parameters in Cancer Patients

The demographic and clinical characteristics of the study population are shown in Table 1. Patients in the NC, BC and RCC groups were subdivided into long and short RTL groups, by applying the median RTL (NC: 1.62; BC: 1.40; RCC: 1.07) as the cut-off value. In the NC population, no significant correlation was observed between RTL and gender (*p* = 0.066) and smoking status (*p* = 0.651). In patients with BC, no significant correlation was observed between RTL and gender (*p* = 0.674), smoking status (*p* = 0.875), histological grading (*p* = 0.582), stage (*p* = 0.178), metastatic disease (*p* = 0.612) or lymphovascular invasion (*p* = 0.074) using the chi-squared test. In patients with RCC, no significant correlation was found between RTL and gender (*p* = 0.601), smoking status (*p* = 1.000), grade (*p* = 0.411), or stage (*p* = 0.717). However, we found that RTL was significantly longer in chromophobe or papillary RCC compared to clear cell RCC (ccRCC; *p* = 0.007).

### 3.3. Correlation between PBL RTL and Overall Survival (OS) in Patients with BC and RCC

**Figure 3:**
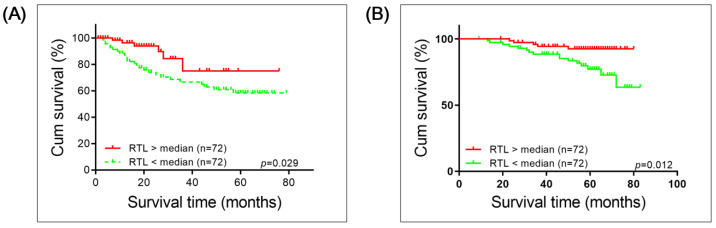
Kaplan–Meier survival analysis of overall survival of patients with (**A**) Bladder Cancer or (**B**) Renal cell carcinoma correlated with long and short relative telomere length (RTL). Significance was determined using log-rank test.

Kaplan–Meier survival analysis with a log-rank test was applied to investigate whether long or short RTL correlates with OS prognosis of patients with BC or RCC. Both groups of patients with short RTL had worse survival compared to those with long RTL (BC: *p* = 0.029; RCC: *p* = 0.012; Figure 3).

In addition, univariate analysis in the BC cohort revealed that muscle invasiveness (*p* < 0.001), metastasis (*p* = 0.001), lymphovascular invasion (*p* = 0.001) and short RTL (*p* = 0.035) were significantly associated with reduced OS, while age (*p* = 0.837), gender (*p* = 0.225), smoking status (*p* = 0.707) and grade (*p* = 0.393) did not significantly correlate with OS. Multivariate analysis in the BC cohort showed that short PBL RTL (*p* = 0.039) is an independent predictive parameter for worse OS, and similarly for muscle invasiveness (*p* = 0.005) and metastasis (*p* = 0.014; Table 2).

In the RCC cohort, univariate analysis indicated that short PBL RTL (*p* = 0.018) and, as expected, tumor stage (*p* = 0.019) were significantly associated with reduced OS. Age (*p* = 0.078), gender (*p* = 0.451), smoking status (*p* = 0.663), tumor grade (*p* = 0.143) and histological subtype (*p* = 0.872) did not significantly correlate with OS of patients with RCC. Similarly, multivariate analysis showed that short PBL RTL (*p* = 0.041) and higher tumor stage (*p* = 0.018) were independent prognostic factors for reduced OS in RCC (Table 2).

## 4. Discussion

In this study, we investigated the relative telomere length (RTL) of peripheral blood leukocyte (PBL) cells of patients with BC or RCC. We demonstrate that shorter TL in PBL cells is associated with worse survival of patients with BC or RCC. Moreover, our findings show that PBL TL is significantly shorter in newly diagnosed BC and RCC patients compared to a normal control population without malignant disease, adding further evidence that PBL TL shortening is associated with progression of BC and RCC.

Numerous studies have focused on the correlation between cell-intrinsic TL and cancer risk. In vivo and in vitro studies over the last 20 years revealed that excessive telomere shortening can lead to dysfunctional telomeres and chromosomal end-to-end fusions and promote tumorigenesis as a result of increased chromosomal instability in checkpoint-deficient cells [35,36,37]. On the other hand, there is evidence indicating an association between cell-intrinsic long telomeres and cancer risk. Recent observations revealed that peripheral blood leukocyte telomere length can serve as a prediagnostic marker for increased cancer risk in solid tissues. Interestingly, some studies found a positive association, while others reported a negative association between leukocyte TL and cancer risk. For instance, human population-based meta-analysis studies (involving nine breast, four bladder, three lung, two kidney, two gastric and two colorectal cancer studies, as well as studies on seven tumor entities of other origin) revealed that shorter PBL telomeres were significantly associated with increased cancer risk, especially in subgroups of BC, but not in RCC [75]. However, based on the UK biobank and TCGA datasets, Gao et al. conducted another study using the genetic risk score (GRS) and Mendelian randomization (MR). The authors revealed that shorter leukocyte TL was associated with decreased risk of developing cancer, including several solid and hematological cancers, e.g., BC, RCC, prostate cancer, multiple myeloma, chronic lymphocytic leukemia, malignant melanoma, basal cell carcinoma, sarcoma and Hodgkin’s lymphoma, among others [76]. On the other hand, Xu et al. found an association between long leukocyte telomere length and increased risk of soft tissue sarcoma [77]. Moreover, Luu et al. demonstrated a significant association between longer leukocyte telomeres and a higher risk of colorectal cancer [78] and pancreatic cancer [79]. Along the same line, Samavat et al. showed evidence that longer telomeres may represent a risk factor for breast [80], rectal, prostate, pancreatic and lung cancer [81]. Interestingly, Wang et al. reported a U-shaped relationship, showing that either extremely long or short PBL TL is associated with higher risk in gastric cancer patients [82].

In the context of bladder cancer, Wang et al. reported that long and heterogeneous TL in blood lymphocytes was strongly associated with an increased bladder cancer risk in an Egyptian population [67]. Another study was performed by Weischer et al., who followed 47,102 individuals for 20 years by prospectively collecting blood samples. In this long-term study, 3142 people developed cancer during the follow-up period, including 131 individuals with cancers of the urinary tract and 59 with kidney cancer. The authors did not observe an association between shorter leukocyte TL and a risk of developing cancer of urinary tract or kidney cancer [83].

However, our results showing shorter TL in the PBL of patients with BC than in control individuals are not in concordance with those two studies, but in line with a study by McGrath et al., who showed that patients with BC had shorter telomeres in PBLs compared to PBLs in control individuals [68]. There are several considerations that can serve as possible explanations for this discrepancy. First, besides the difference in ethnicities included in the Wang et al. study, the authors determined TL by telomere quantitative fluorescent in situ hybridization (TQ-FISH) to measure the average relative telomere length. Second, the authors determined telomere length variation (TLV) as a second parameter and combined it with TL, showing that long TL with high TLV represented a 14-fold difference compared to short TL and low TLV. Thus, using a different method and a different group setting may lead to a different outcome and conclusion. Finally, the Weischer et al. study included tumors from the whole urinary tract. Considering the low number of samples for the whole urinary tract in this study, it is not clear whether their data are comparable to our results for BC. In the case of RCC, our data are in line with the report by Shao et al. indicating that telomere shortening in peripheral blood lymphocytes might be a genetic predisposing factor for cancer [69]. However, using a large meta-analysis study assessing 10,784 RCC cases and 20,406 cancer-free controls from six genome-wide association studies (GWAS), Machiela et al. reported that genetic variants related to longer PBL TL are strongly associated with increased risk for developing RCC [70]. Thus, whether shortening or lengthening of PBL telomeres is a predictive risk factor for developing RCC remains an open question. Regarding our results showing that the papillary and chromophobe RCC group display significant longer RTL, we still keep cautiously optimistic due to the small sample numbers. Further studies and larger cohorts are needed to draw a more clear conclusion.

An increasing number of studies indicate that leukocyte TL might be a prognostic parameter for the survival of patients with different types of cancer. For instance, Svenson et al. found that long leukocyte TL correlated with worse survival in breast cancer [58], ccRCC [59] and prostate cancer [60], which was also observed in hepatocellular carcinoma by Liu et al. [61]. Other studies revealed that short leukocyte TL was associated with worse survival in BC [62], gastric cancer [63], colorectal cancer [64,65], ovarian cancer and cervical cancer [66]. Using GRS and MR approaches, Gao et al. demonstrated that decreased leukocyte TL was associated with worse overall survival of patients with rectum adenocarcinoma, sarcoma and skin cutaneous melanoma, but better overall survival of patients with papillary RCC [76].

Our findings show that short PBL TL correlates with worse survival of patients with BC or RCC as an independent factor for survival. As expected, we also observed that higher stages/muscle invasiveness and metastasis in BC and higher stages in RCC are independent negative factors for the overall survival of patients. With regard to the correlation between PBL TL and overall survival of patients with BC (Figure 3 and Figure S3), our results are consistent with those of Russo et al. [62]. In agreement with our results, they also did not observe an association between relative PBL TL and tumor stage (Tis, Ta, T1 BC as NMIBC/T2 and higher stages as MIBC) or tumor grade (HG/NHG). In our study, we included a heterogeneous patient cohort to cover up distinct tumor characteristics, comprising subgroups with distinct prognosis. As an example, patients undergoing transurethral resection (TURB) for superficial BC (low or high grade) will have better survival outcomes compared to patients undergoing radical cystectomy for invasive BC. However, patients treated with TURB may have a higher risk of recurrence. It is therefore difficult to define the best outcome parameter in such mixed patient cohorts and we decided to correlate with robust OS data, although this may miss some cancer-specific characteristics.

Tumor grading was not significantly associated with OS in our study cohort. Grading may be less influential on survival in such mixed cohorts, and T staging is a more relevant prognostic factor, However, this may depend on the tumor type. Nevertheless, our retrospective study design including a limited number of patients and follow-up period may also present a bias for outcome measures.

For RCC, in line with our results, Callahan et al. also reported that shorter PBL TL was associated with poorer RCC disease-specific survival [71]. Similarly, results are presented by Chen et al., who showed that short leukocyte TL, as evaluated by GRS, is associated with worse prognosis in RCC patients, supporting our observations [84]. We also analyzed the overall survival of patients in terms of the correlation between PBL TL and histological subtypes, i.e., clear cell, papillary and chromophobe RCC. Notably, the subgroup analysis of ccRCC, the most common type of RCC, showed significantly worse overall survival correlated with shorter PBL TL as an independent factor (Figure S4; Tables S2 and S3). However, Svenson et al. reported that ccRCC patients with the longest blood cell telomeres (fourth quartile) had a significantly worse prognosis (*p* = 0.005), and long blood cell telomeres were found to be significantly associated with worse outcome only in nonmetastatic ccRCC [59]. The reason for this discrepancy may be differences in cohort sizes and tumor stages. Of note, Morais and colleagues suggested that TL may have a dual role in RCC, specifically that short TL could increase RCC risk and cancer progression in late carcinogenesis, while long telomeres may be associated with tumor prognosis in early stages [85]. The relationship between PBL TL and RCC risk and the survival of patients with RCC still remains to be further clarified in future studies.

In summary, our results indicate that RTL of PBL cells is associated with reduced overall survival of patients with BC and RCC (cohorts comprising 144 patients in each group). The results provide evidence that measuring TL in PBL cells may be essential for prediction as a prognostic marker for OS of patients with BC and RCC. How can these results be integrated into the risk assessment of patients? In the light of several studies indicating leukocyte telomere length shortening after chemotherapy of patients with different origins of cancer and the observation that telomere shortening impairs immune response capacity [86,87,88,89,90]. TL determination may provide a supportive information for the choice of chemotherapy for individual, personalized treatment approaches. It is conceivable that patients with shorter TL may have a worse tolerance and higher leukocyte toxicity to certain chemotherapeutic treatments than patients with longer telomeres, who may have PBLs with higher proliferation capacity, less-impaired immune response due to chemotherapy and thus better prognosis. It is worth mentioning that PB was collected before surgery and any therapy in our study group.

The mechanisms of the relationships between leukocyte telomeres and mortality of patients with solid tumors and the risk of developing different types of cancers are not clearly understood. There is evidence that leukocyte telomere length is correlated with environmental factors such as stress, smoking, obesity and socioeconomic group, likely due to increased cell turnover in response to oxidative stressors and injury [91,92,93]. The association between smoking, one of the major risk factors for BC [94] and RCC [95], and mortality could not be revealed in our investigations. This result is in line with a previous report by Russo et al., who also could not find any association between smoking and mortality rate of patients with BC [62].

Telomere length from human PBL is inversely correlated with age [96] and could be influenced by genetic factors [97,98]. Several studies suggest that a higher proliferation rate of immune cells accounts for the association of shorter TL with poorer overall survival. One mechanism could involve regulation of telomere length by regulating telomerase activity via serum factors such as cytokine TGFß1 [65] or interleukins [66], which are known to be regulators of telomerase [99,100]. For instance, a lower concentration of plasma transforming growth factor-β1 (TGF-β1) was correlated with shorter TL in CD4 + T cells [65]. It is also possible that individuals with long leukocyte telomeres carry modifier mutations in telomere length control factors that contribute to tumor initiation. In line with this, individuals with mutations in the shelterin protein Pot1 have longer telomeres at birth and increased risk of developing melanomas [101,102]. We previously provided experimental evidence that telomerase heterozygous (mTerc +/−) BALBc mice with inherent *Prkdc* mutations with long telomeres have shorter tumor latency compared to mice with late generation mTerc-G3 with shorter telomeres [103]. Moreover, single nucleotide polymophisms (SNPs) in telomerase components can result in higher levels of telomerase activity [104,105], potentially promoting tumorigenesis. The complex regulation of TL in PBL cells by genetic or epigenetic factors and the relationship of solid tumors and cancer progression remain to be further elucidated.

Alternatively, based on the observation that immunosuppressive regulatory T cells (Tregs) show a positive correlation with leukocyte TL, Svenson et al. suggested that a subgroup of cancer patients with longer TL might have a less active or more suppressed immune system, with fewer cell divisions and less telomere shortening [106]. This “immunohypothesis” idea could explain the association between longer TL and cancer risk/mortality. Similar observations were reported by Liu et al. in patients with hepatocellular carcinoma [61], where long leukocyte TL corroborated with worse survival and a significantly increased percentage of Tregs.

## 5. Conclusions

In summary, we demonstrate that telomere lengths in PBL cells of patients with BC and RCC are significantly shorter compared with the NC population. Importantly, shorter PBL TL is associated with worse overall survival. The results indicate that PBL TL might be a useful prognostic biomarker to predict the survival of patients with BC or RCC. Further prospective studies with larger patient cohorts are needed to understand the detailed mechanisms.

## Figures and Tables

**Table 1:** Demographic and clinical characteristics of study population (bladder cancer and renal cell carcinoma).

**Control Copulation**				
	**N**	**Long RTL**	**Short RTL**	***p*-Value**
Cohorts	73 (100%)	36 (49.32%)	37 (50.68%)	
Age, mean (SD)	60 (12.61)	58 (12.84)	61 (12.31)	0.019 ^a^
Gender				
Male	59 (80.82%)	26 (44.07%)	33 (55.93%)	
Female	14 (19.18%)	10 (71.43%)	4 (28.57%)	0.066 ^b^
Smoking status				
No smoking	53 (72.60%)	27 (50.94%)	26 (49.06%)	
Smoking	20 (27.40%)	9 (45%)	11 (55%)	0.651 ^b^
**Bladder Cancer**				
	**N**	**Long RTL**	**Short RTL**	***p*-Value**
Patients	144 (100%)	72 (50%)	72 (50%)	
Age, mean (SD)	68 (10.94)	67 (10.08)	69 (11.75)	0.036 ^a^
Gender				
Male	116 (80.56%)	57 (49.14%)	59 (50.86%)	
Female	28 (19.44%)	15 (53.57%)	13 (46.43%)	0.674 ^b^
Smoking status				
No smoking	99 (68.75%)	49 (49.49%)	50 (50.51%)	
Smoking	45 (31.25%)	23 (51.11%)	22 (48.89%)	0.875 ^b^
Grade				
NHG	33 (22.92%)	15 (45.45%)	18 (54.55%)	
HG	108 (75%)	55 (50.93%)	53 (49.07%)	0.582 ^b^
Muscle invasiveness				
NMIBC (Tis, Ta, T1)	82 (56.94%)	45 (54.88%)	37 (45.12%)	
MIBC (T2, 3, 4)	62 (43.06%)	27 (43.55%)	35 (56.45%)	0.178 ^b^
Metastasis				
No	140 (97.22%)	71 (50.71%)	69 (49.29%)	
Yes	4 (2.78%)	1 (25%)	3 (75%)	0.612 ^c^
Lymphovascular invasion (LVI)				
No	120 (83.33%)	64 (53.33%)	56 (46.67%)	
Yes	24 (16.67%)	8 (33.33%)	16 (66.67%)	0.074 ^b^
**Renal Cell Carcinoma**				
	**N (%)**	**Long RTL (%)**	**Short RTL (%)**	***p*-Value**
Patients	144 (100)	72 (50%)	72 (50%)	
Age, mean (SD)	69 (10.72)	65 (11.16)	73 (8.33)	<0.001 ^a^
Gender				
Male	93 (65.28%)	48 (51.61%)	45 (48.39%)	
Female	51 (35.42%)	24 (47.06%)	27 (52.94%)	0.601 ^b^
Smoking status				
No smoking	122 (84.72%)	61(50%)	61 (50%)	
Smoking	22 (15.28%)	11 (50%)	11 (50%)	1.000 ^b^
Grade				
grade 1, 2	92 (65.25%)	48 (52.17%)	44 (47.83%)	
grade 3	49 (34.75%)	22 (44.90%)	27 (55.10%)	0.411 ^b^
Stage				
T 1,2	100 (69.44%)	51 (51%)	49 (49%)	
T 3	44 (30.56%)	21 (47.73%)	23 (52.27%)	0.717 ^b^
Pathology types				
ccRCC	120 (83.33%)	54 (45%)	66 (55%)	
Other types	24 (16.67%)	18 (75%)	6 (25%)	0.007 ^b^

The NHG (non-high grade) BC refers to the cases with G1 and G2 grades (WHO/1973) and low-grade (WHO/2004) bladder cancer. The HG (high-grade) BC consists of cases with G3 (WHO/1973) and high-grade (WHO/2004) bladder cancer. NMIBC, non-muscle-invasive bladder cancer; MIBC, muscle-invasive bladder cancer; RTL, relative telomere length. ^a^ *p*-value was calculated using Spearman′s correlation analysis; ^b^ *p*-value was calculated using Pearson’s chi-squared test; ^c^ *p*-value was calculated using Pearson’s chi-squared test with Yates′s correction for continuity.

**Table 2:** Univariate and multivariate Cox regression survival analysis of prognostic factors of bladder cancer and renal cell carcinoma. RTL, relative telomere length.

**Bladder Cancer**							
**Factors**	**Cases**	**Univariate Analysis**			**Multivariate Analysis**		
		**HR**	**95% CI**	***p*-Value**	**HR**	**95% CI**	***p*-Value**
Age							
≤68	77	1.08	0.528–2.200	0.837	NA	NA	NA
>68	67						
Gender							
Male	116	1.60	0.750–3.396	0.225	NA	NA	NA
Female	28						
Smoking status							
No smoking	99	0.86	0.396–1.873	0.707	NA	NA	NA
Smoking	45						
Grade							
NHG	33	1.52	0.581–3.980	0.393	NA	NA	NA
HG	108						
Muscle invasiveness							
NMIBC	82	5.01	2.054–12.215	<0.001	4.036	1.528–10.658	0.005
MIBC	62						
Metastasis							
No	140	8.74	2.520–30.337	0.001	4.995	1.385–18.009	0.014
Yes	4						
Lymphovascular invasion							
No	120	3.31	1.583–6.912	0.001	1.499	0.671–3.351	0.324
Yes	24						
RTL							
long	72	2.65	1.071–6.562	0.035	2.613	1.049–6.510	0.039
short	72						
**Renal Cell Carcinoma**							
**Factors**	**Cases**	**Univariate Analysis**			**Multivariate Analysis**		
		**HR**	**95% CI**	***p*-Value**	**HR**	**95% CI**	***p*-Value**
Age							
≤69	71	2.34	0.908–6.305	0.078	1.59	0.592–4.268	0.357
>69	73						
Gender							
Male	93	0.69	0.269–1.794	0.451	NA	NA	NA
Female	51						
Smoking status							
No smoking	122	1.27	0.428–3.790	0.663	NA	NA	NA
Smoking	22						
Grade							
Grade 1, 2	92	1.92	0.801–4.608	0.143	NA	NA	NA
Grade 3	49						
Stage							
T 1,2	100	2.80	1.188–6.590	0.019	2.809	1.192–6.623	0.018
T 3	44						
Pathology types							
ccRCC	120	0.90	0.264–3.097	0.872	NA	NA	NA
Other types	24						
RTL							
long	72	3.36	1.231–9.183	0.018	2.913	1.021–8.310	0.046
short	72						

NHG, non-high-grade; HG, high-grade; NMIBC, non-muscle-invasive bladder cancer; MIBC, muscle-invasive bladder cancer; RTL, relative telomere length. Other types of RCC include papillary and chromophobe RCC; NA, not applicable.

## Data Availability

The data presented in this study are available in this article and the supplementary materials.

## Supplementary Materials

The following data are available online at https://www.mdpi.com/article/10.3390/cancers13153774/s1, Figure S1: Control experiments; Figure S2: Comparison of RTL; Figure S3: Kaplan-Meier survival analysis of BC overall survival in correlation with long and short RTL groups; Figure S4: Kaplan-Meier survival analysis of patients with ccRCC in correlation with long and short RTL groups; Table S1: Buffers for DNA ex-traction; Table S2: Demographic and clinical characteristics of the ccRCC subgroup population; Table S3: Univariate and multivariate cox regression survival analysis of prognostic factors of ccRCC.
