# Supplementary material for: Shorter Leukocyte Telomere Length Is Associated with Worse Survival of Patients with Bladder Cancer and Renal Cell Carcinoma
Source: Cancers (Basel). 2021 Jul 27;13(15):3774. doi: 10.3390/cancers13153774 (PMC8345040; doi:10.3390/cancers13153774)
Supplement: Supplementary file 1 [file cancers-13-03774-s001.zip › cancers-1227577-supplementary.pdf]

cancers

MDPI

Supplementary Materials

Table S1. Buffers for DNA extraction

**lysis buffer** (PH 7.4)	
NH_4Cl	16.58 g
$KHCO_3$	2.0 g
$(Na_2)EDTA$	400 µl 0.5 M
diluted in 2 l sterile H_2O	

**SE buffer** (PH 7.4)	
NaCl	1.097 g
$(Na_2)EDTA$	2.1 g
diluted in 250 ml sterile H_2O	

**TE buffer** (PH 7.4)	
Tris	0.242 g
$(Na_2)EDTA$	0.0744 g
diluted in 200 ml sterile H_2O	

20% SDS	20 g/100 ml sterile H_2O

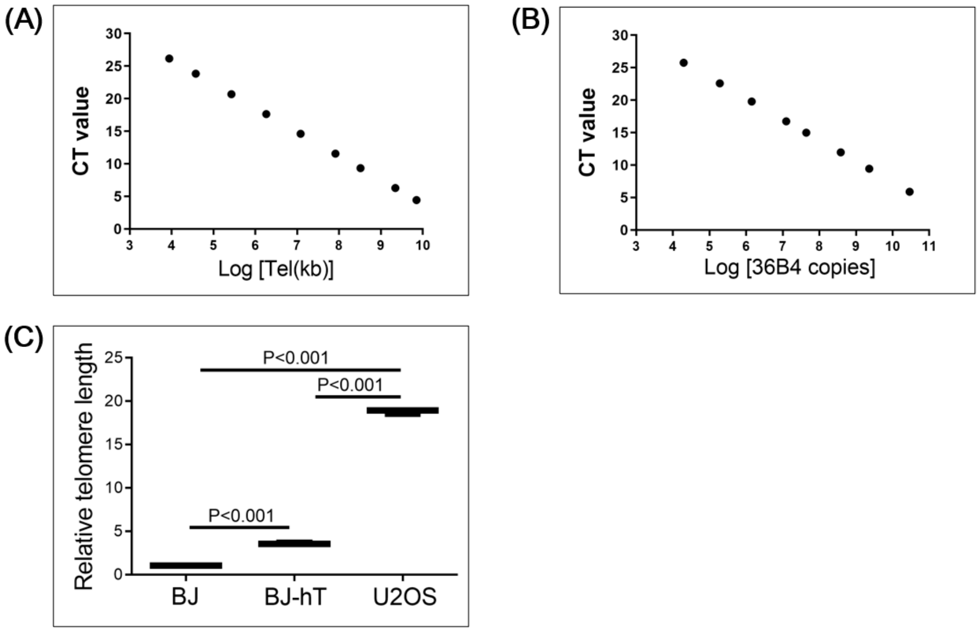

Figure S1. Control experiments. The standard curve was used to measure the telomere DNA length per sample in kb for telomere standard and diploid genome copies in relation to the human 36B4 DNA copy number. RTL of BJ, BJ-hTERT and U2OS cells were used to confirm the experimental conditions. (**A**) Standard curve for calculating length of telomeric DNA. X-axis represents amount of telomere sequence in kb per reaction; (**B**) Standard curve for calculating genomic copies in relation to 36B4 copy number; (**C**) Relative telomere length of BJ, BJ-hTERT and U2OS cells (the absolute TL from BJ cells were set as baseline: 1).

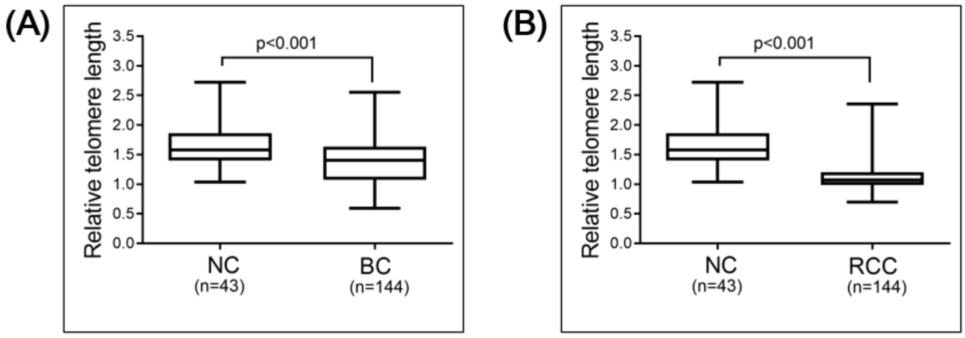

Figure S2. Comparison of RTL: (**A**) NC group (n = 43) versus BC group (n = 144); (**B**) NC group (n = 43) versus RCC group (n = 144). Comparisons were performed using Mann–Whitney test. NC, normal control; BC, bladder cancer; RCC, renal cell carcinoma.

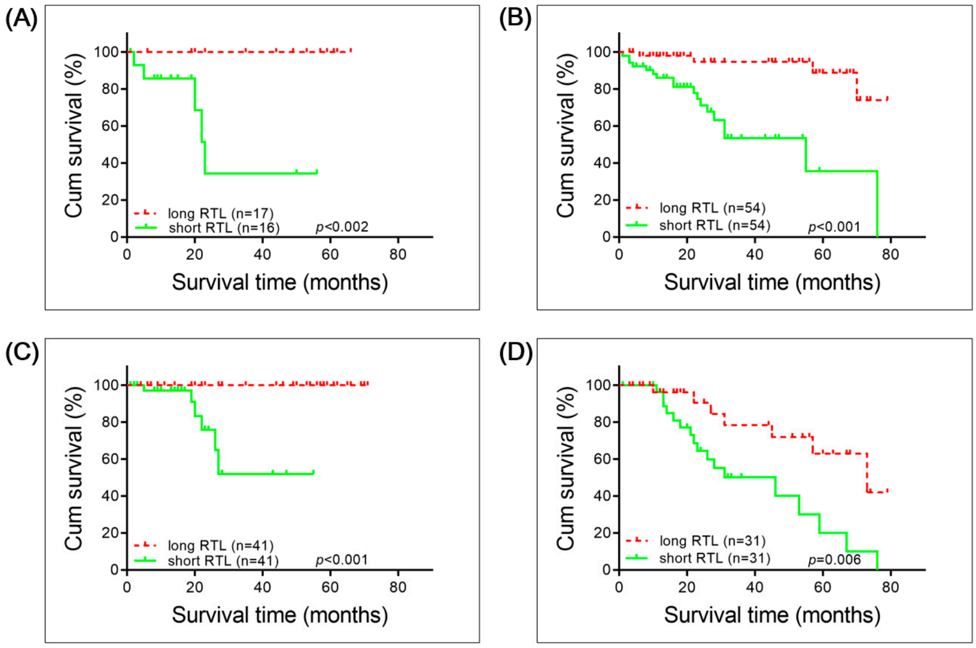

Figure S3. Kaplan-Meier survival analysis of BC overall survival in correlation with long and short RTL groups: (A) Overall survival of NHG BC patients; B) overall survival of HG BC patients; (C) overall survival of NMIBC patients; D) overall survival of MIBC patients. NHG: Non-high grade; HG: high grade; NMIBC: Tis, Ta, T1 bladder cancer; MIBC: T2 and higher stages bladder cancer.

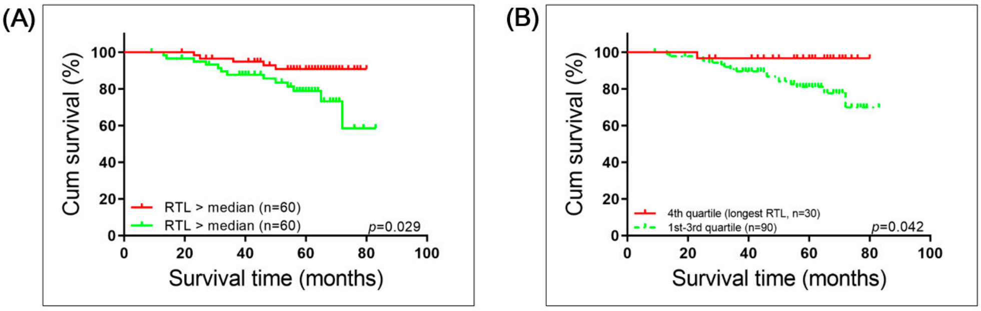

Figure S4. Kaplan-Meier survival analysis of patients with ccRCC in correlation with long and short RTL groups: ccRCC patients with shorter RTL had significantly worse survival. (A) Two groups were stratified with median RTL; (B) the longest RTL quartiles versus first to third short RTL quartiles.

Table S2. Demographic and clinical characteristics of the ccRCC subgroup population

ccRCC				
	**N(%)**	**long RTL(%)**	**short RTL(%)**	***p* value**
Patients	120 (100)	60 (50%)	60 (50%)	
Age,mean(SD)	70 (10,08)	65.8 (10.09)	73.93 (8.34)	**<0.001**[a]
Gender				
Male	77 (64.17%)	41 (53.25%)	36 (46.75%)	
Female	43 (35.83%)	19 (44.19%)	24 (55.81%)	0.314[b]
Smoking status				
No smoking	104 (86.67%)	48 (46.15%)	56 (53.85%)	
Smoking	16 (13.33%)	6 (37.5%)	10 (62.5%)	0.517[b]
Grade				
grade 1,2	79 (67.52%)	43 (54.43%)	36 (45.57%)	
grade 3	38 (32.48%)	17 (44.74%)	21 (55.26%)	0.326[b]
Stage				
T 1,2	85 (70.83%)	43 (50.59%)	42 (49.41%)	
T 3	35 (29.17%)	17 (48.57%)	18 (51.43%)	0.841[b]

N,number of cases; SD, standard deviation; RTL, relative telomere length; a, the p value was calculated using Spearman's correlation analysis; b, the p value was calculated using Pearson chi square test.

Table S3. Univariate and multivariate cox regression survival analysis of prognostic factors of ccRCC

ccRCC								
**Factors**	**Cases**	**Univariate Analysis**			**Multivariate Analysis**			
		**HR**	**95%CI**	**p value**	**HR**	**95%CI**	**p value**
Age							
≤69	60	1.96	0.736-5.229	0.178	NA	NA	NA
>69	60						
Gender							
Male	77	0.48	0.159-1.473	0.201	NA	NA	NA
Female	43						
Smoking status							
No smoking	104	1.82	0.597-5.540	0.293	NA	NA	NA
Smoking	16						
Grade							
Grade1,2	79	1.85	0.708-4.808	0.21	NA	NA	NA
Grade 3	38						
Stage							
T 1,2	85	2.70	1.069-6.791	**0.036**	2.782	1.102-7.019	**0.03**
T 3	35						
RTL							
long	60	2.99	1.064-8.415	**0.038**	3.074	1.094-8.638	**0.033**
short	60						

RTL, relative telomere length. ccRCC, clear cell renal cell carcinoma; NA, not applicable.
